# Cellular senescence in the aging retina and developments of senotherapies for age-related macular degeneration

**DOI:** 10.1186/s12974-021-02088-0

**Published:** 2021-01-22

**Authors:** Keng Siang Lee, Shuxiao Lin, David A. Copland, Andrew D. Dick, Jian Liu

**Affiliations:** 1 Bristol Medical School, Translational Health Sciences, University of Bristol, Bristol, BS8 1TD UK; 2 School of Cellular and Molecular Medicine, University of Bristol, Bristol, BS8 1TD UK; 3 Institute of Ophthalmology, University College London, London, EC1V 9EL UK; 4 National Institute for Health Research Biomedical Research Centre, Moorfields Eye Hospital, London, EC1V 2QH UK

**Keywords:** Macular degeneration, Cellular senescence, SASP, Immune aging, Retinal pigment epithelium, Microglia, Neuron

## Abstract

Age-related macular degeneration (AMD), a degenerative disease in the central macula area of the neuroretina and the supporting retinal pigment epithelium, is the most common cause of vision loss in the elderly. Although advances have been made, treatment to prevent the progressive degeneration is lacking. Besides the association of innate immune pathway genes with AMD susceptibility, environmental stress- and cellular senescence-induced alterations in pathways such as metabolic functions and inflammatory responses are also implicated in the pathophysiology of AMD. Cellular senescence is an adaptive cell process in response to noxious stimuli in both mitotic and postmitotic cells, activated by tumor suppressor proteins and prosecuted via an inflammatory secretome. In addition to physiological roles in embryogenesis and tissue regeneration, cellular senescence is augmented with age and contributes to a variety of age-related chronic conditions. Accumulation of senescent cells accompanied by an impairment in the immune-mediated elimination mechanisms results in increased frequency of senescent cells, termed “chronic” senescence. Age-associated senescent cells exhibit abnormal metabolism, increased generation of reactive oxygen species, and a heightened senescence-associated secretory phenotype that nurture a proinflammatory milieu detrimental to neighboring cells. Senescent changes in various retinal and choroidal tissue cells including the retinal pigment epithelium, microglia, neurons, and endothelial cells, contemporaneous with systemic immune aging in both innate and adaptive cells, have emerged as important contributors to the onset and development of AMD. The repertoire of senotherapeutic strategies such as senolytics, senomorphics, cell cycle regulation, and restoring cell homeostasis targeted both at tissue and systemic levels is expanding with the potential to treat a spectrum of age-related diseases, including AMD.

## Background

The steady increase in life expectancy and the aging population has presented us with a new challenge: age-related diseases. Age-related macular degeneration (AMD), a degenerative disease affecting the central area of the neuroretina (macula) and supporting retinal pigment epithelium, is the leading cause of registered blindness in western countries in those aged 65 and over [1, 2]. It is steadily becoming a major public health issue as the global AMD burden is projected to reach 288 million people by 2040 [1, 2]. In the USA, approximately 11 million people are affected by AMD, a prevalence that is similar to that of all invasive cancers combined, and over double of that of Alzheimer’s disease (AD) [1]. Visual loss significantly affects activities of daily living, and people fear losing sight as much as developing cancer or dementia [3]. The global cost of visual impairment due to AMD alone is substantial, estimated to be US$343 billion including 74% in direct healthcare costs (AMD Alliance International).

AMD is a progressive, polygenic, and multifactorial disease with complex etiology. Although age remains the primary risk factor for AMD, the interplays of susceptible genes associated with complement activation, lipid metabolism, cholesterol transport, receptor-mediated endocytosis, and extracellular matrix organization, alongside environmental risk factors, such as smoking, diet, obesity, sunlight exposure, alcohol consumption, and cardiovascular disease, increase the risk of developing or are associated with enhanced severity of AMD [4–6]. Clinically, early AMD can be characterized by the deposition of lipoproteinaceous drusen at the sub-retinal pigment epithelium (RPE) accompanied by pigmentary abnormalities in the RPE, which progresses into two late forms, dry (atrophic) and wet (neovascular) [1, 7]. Dry AMD is a slow progressive deterioration in visual function characterized by insidious atrophy of the RPE and rod and cone photoreceptors [1, 2]. Ten to 15% of patients with dry AMD progress to the wet form, distinguished by the generation of an abnormal choroidal neovascular membrane into the macula (a process termed as choroidal neovascularization or CNV), which is prone to leakage or hemorrhage beneath the retina leading to retinal scarring and sudden vision loss [1, 2]. Current anti-vascular endothelial growth factor (VEGF) therapies have revolutionized treatment for the inhibition of the pathological neoangiogenesis of wet AMD (advanced disease), however, many years after the progression of degeneration has ensued [8, 9]. Due to the lack of treatment to counter the degenerative processes per se, a substantial proportion of AMD eventually leads to severe visual impairment or blindness [1, 7].

Despite our increased understanding, active therapeutic intervention remains of limited benefit for patients with AMD. The Age-Related Eye Disease Studies (AREDS/AREDS2) assessed the effects of nutritional supplements on the course of AMD and found that taking antioxidant nutrient formula (vitamin C, vitamin E, lutein, and zeaxanthin) in combination with zinc can reduce the progression from intermediate to advanced AMD by 25%, however, with no effect in preventing early AMD [10, 11]. Current clinical trials investigating drugs, biologics, and small molecules to target the biological pathways in AMD, such as oxidative stress, visual cycle inhibition, complement activation, retinal/choroidal blood flow, amyloid beta (Aβ), and lipid accumulation, have yet to generate tractable therapies [12, 13].

The eye is an ideal organ for gene therapy because of the relative ease of access and compartmentalization, relative immune privilege, and small size reducing the viral load required. Unsurprisingly, the use of ocular gene therapy has been successful in a variety of ocular diseases [14, 15]. These include gene therapy trials for inherited retinal degenerations, such as Leber congenital amaurosis (LCA), retinitis pigmentosa, choroideremia, and X-linked retinoschisis (XLRS) [14, 16, 17]. As reported, seven gene therapy trials have been developed to treat wet AMD via transgene infections to continuously express antiangiogenic proteins, such as anti-VEGF Fab, soluble fms-like tyrosine kinase-1 (sFlt-1), and endostatin [18, 19]. For dry AMD, there are currently two ongoing gene therapy trials, including GT005 (phase I) that induces ocular complement factor I (CFI) expression [20] and HMR59 (AAVCAGsCD59, phase I) that expresses C59 to prevent formation of the membrane attack complex (MAC) [21].

The goal to prevent the progressive loss of RPE and photoreceptors that characterize the macular degeneration relies on our understanding of key events defining the transition from normal aging to AMD. Altered pathways and processes of cellular metabolism, oxidative stress, cellular senescence, and associated inflammatory responses are all potential contributory or causative factors disrupting tissue homeostasis [22–24]. Cellular senescence is one of the adaptive cell responses induced by different types of stress and accompanying normal aging [23]. Disproportionate senescent changes in tissue-resident cells or systemic immune cells exacerbate the adverse effects of aging, which clinically lead to different chronic disorders, including AMD [25–27]. This review strives to provide current insights of cellular senescence in retinal aging and development of AMD, over and above a perspective of senotherapeutic strategies against AMD to achieve a healthy aging with vision.

## Senescence is an adaptive and programmed cellular response with distinct characteristics

The concept of senescence, the condition or process of gradual deterioration with age, was formulated by Peter Medawar in the 1950s [28]. During the early 1960s, Leonard Hayflick and Paul Moorhead further ascribed the senescence at the cellular level as a state of irreversible cessation of cell division when the cells entered the end of their replicative lifespan but remained viable [29]. Cellular senescence is a cell-intrinsic and adaptive response to a variety of exogenous and endogenous stimuli, originally characterized by a stable cell cycle arrest (G1 arrest) only in mitotic cells [30, 31]. Recent experimental and clinical evidence reveals senescence in postmitotic and rare mitotic cells, such as the terminally differentiated neurons of the central nervous system (CNS), cardiomyocytes, osteocytes, and adipocytes [32–34]. Regarded as a multistep process, the initiation and induction of senescence in the cells requires the activation of tumor suppressors involved in the p53/p21^CIP1^ and p16^INK4A^/retinoblastoma protein (RB) pathways [31, 35]. Another key feature of cellular senescence is the senescence-associated secretory phenotype (SASP) wherein the senescent cells release a host of proinflammatory mediators through pathways dependent on p38 MAPK, NF-κB, Notch, and/or mTOR signaling [36–38]. Cellular senescence is operative both physiologically and pathologically, ranging from embryonic development, tissue remodeling, wound repair, cancer, and aging [30–32].

Similar to apoptosis and autophagy, cellular senescence is a distinct cell response to intrinsic cues including telomere shortening, metabolic and proteostatic dysfunction, as well as extrinsic factors such as increased oxidative stress incited by UV radiation, cigarette smoking or microbial infections, and chronic stress from inflammation [39]. Cellular senescence is also attributed to other forms of genotoxic stress, mitogens or inflammatory cytokines, that culminate in the activation of the p53 tumor suppressor and/or the cyclin-dependent kinase inhibitor p16^INK4A^ [39]. Cellular senescence can be classified into three types: replicative senescence (RS), stress-induced premature senescence (SIPS), and developmentally programmed senescence (DPS) [31]. RS is mainly induced by telomere shortening or dysfunction, whereas SIPS can be telomere independent [37].

A physiological role of programmed cellular senescence is to maintain normal tissue homeostasis including tissue repair and tumor suppression [35, 40, 41]. Cells including myofibroblasts enter acute senescence to reduce fibrosis [42, 43], and with defective senescence (e.g., deficiency in p53, p16^INK4A^, or TRE-shp53), excessive fibrosis ensues in the liver [42]. When there is expression of transcription factors OCT4, SOX2, KLF4, and MYC (OSKM), reprogramming of a small population of adult-differentiated cells into the pluripotent stem cells occurs with an induction of senescence of many other cells. The OSKM-driven senescent cells promote cell reprogramming that facilitates tissue repair [41]. Similarly in tumors, oncogene-induced senescence has been regarded as an intrinsic mechanism for tumor suppression and immune surveillance [40]. Loss of key senescence-inducing genes—*p53*, *p16*^*INK4A*^*,* or *p19*^*ARF*^—causes an inactivation of the senescence pathways favoring malignant transformation [44].

Senescent cells undergo distinctive and profound morphological and functional alterations in chromatin, metabolism and secretome, both in vitro and in vivo [31, 35, 45]. These include (1) altered cellular morphology (often enlarged, flat, multivacuolated, and multinucleated); (2) increased senescence-associated *β*-galactosidase (SA-*β*-Gal) activity; (3) the accumulation of DNA damage foci including double-strand breaks (DSBs); (4) the accumulation of chromatin modifications, such as senescence-associated heterochromatic foci (SAHF) that is enriched in a transcription-silencing histone H2A variant (macroH2A); (5) chromosomal instability associated with the reduction in lamin B1 and the release of high mobility group box 1 protein (HMGB1); (6) altered mitochondrial metabolism with elevated glycolytic rate; (7) a SASP phenotype producing cytokines, growth factors, proteases, and lipids, which constitutes a senescence-specific micro-environment influencing surrounding cells [46, 47]; and (8) the expression of senescence marker proteins (i.e., p38 MAPK, p53, p21^CIP1^, p16^INK4A^, RB, and cyclin-dependent kinases (CDKs)) [37]. These characteristics might reflect either the causal factors or consequences of senescence. These non-exclusive features could also be the by-products of physiological changes in non-replicating cells or non-senescent cells [37, 48]. A robust and unequivocal panel of markers of the senescence has to be determined in a cell population-dependent context [37, 48]. Moreover, subsets of senescent cells with phenotypic and functional heterogeneity are recognized and must be considered for therapeutic targeting [49].

## Cell senescence is an important driver for age-related diseases including AMD

**Fig. 1:**
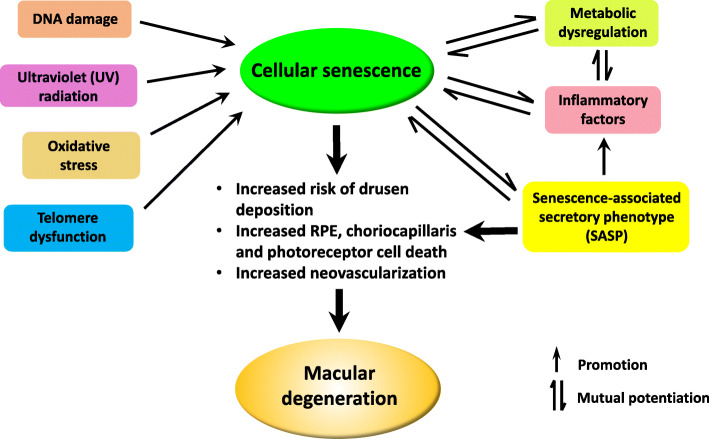
Etiology and consequences of cellular senescence contributing to AMD. Cellular senescence is highly relevant to AMD pathogenesis. A variety of factors—telomere dysfunction, oxidative stress, nutrient signals, DNA damage, and inflammatory cytokines—can activate cellular senescence in both mitotic and post-mitotic cells. Senescent cells display altered metabolic function and autophagy activity, as well as a distinctive proinflammatory secretome, which are all interlinked. These processes directly impose a cause-and-effect on one another, further accelerating their progression. The consequences of chronic cellular senescence, such as increased drusen deposition, increased RPE, choriocapillaris and photoreceptor dysfunction and cell loss, and/or neovascularization, ultimately lead to the advanced disease phenotype of macular degeneration

As the protective role of senescence relies on acute response to adverse molecular damages combined with the timely elimination of senescent cells via cell death or immune actions, overproduction of senescent cells or impairment of the removal mechanism will lead to a detrimental hoarding of senescent cells, termed as “chronic” senescence [43, 50]. Age-associated changes in the cells, such as telomere erosion, DNA lesions, ROS production, and metabolic disturbance are potent triggers that may induce senescence. The persistent accumulation of senescent cells due to chronic stress is a result of aging and a major driver of age-related illnesses [45]. Examples are pulmonary fibrosis, diabetic pancreas, osteoarthritis, atherosclerosis, AD, and Parkinson’s disease (PD), as well as ocular diseases such as glaucoma and cataracts [51]. The senescent cell burden in the brain induces inflammation and alters iron metabolism, changes associated with neurodegeneration and cognitive decline in an AD model. Conversely, elimination of senescent cells mitigates the disease severity [52]. As in other adult tissues, the senescence program may be elicited in the eye by various exogenous and endogenous factors such as oxidative stress, metabolic disturbance, nutrient and growth factor signals, telomere dysfunction, and DNA damage [53–55]. Other stimulants including autophagy dysfunction and abnormal inflammatory response also play a role in activating cellular senescence (Fig. 1).

Cumulative evidence has demonstrated senescent RPE cells in human AMD donor eyes and old non-human primate eyes [56–59]. In addition to RPE, retinal neurons, choroidal endothelial cells, and retinal microglia also demonstrate senescence in association with the retinal aging and/or pathogenesis of AMD [47, 60, 61]. As unveiled in a senescence-accelerated OXYS rat model, the retina exhibits transcriptome changes in genes involved in pathways associated with inflammation, apoptosis, DNA damage, and oxidative stress, and develop pathologic changes akin to human AMD [62]. As one of the central mediators for both RS and SIPS, age-associated damages in nuclear DNA (nDNA) and mitochondrial DNA (mtDNA), alongside a declined capacity of DNA repair, together have strong clinical associations with both atrophic and neovascular AMD [63, 64]. In aggregate, the data purport that enhanced cellular senescence drives disease progression.

## Senescence occurs in a variety of retinal cells

**Fig 2:**
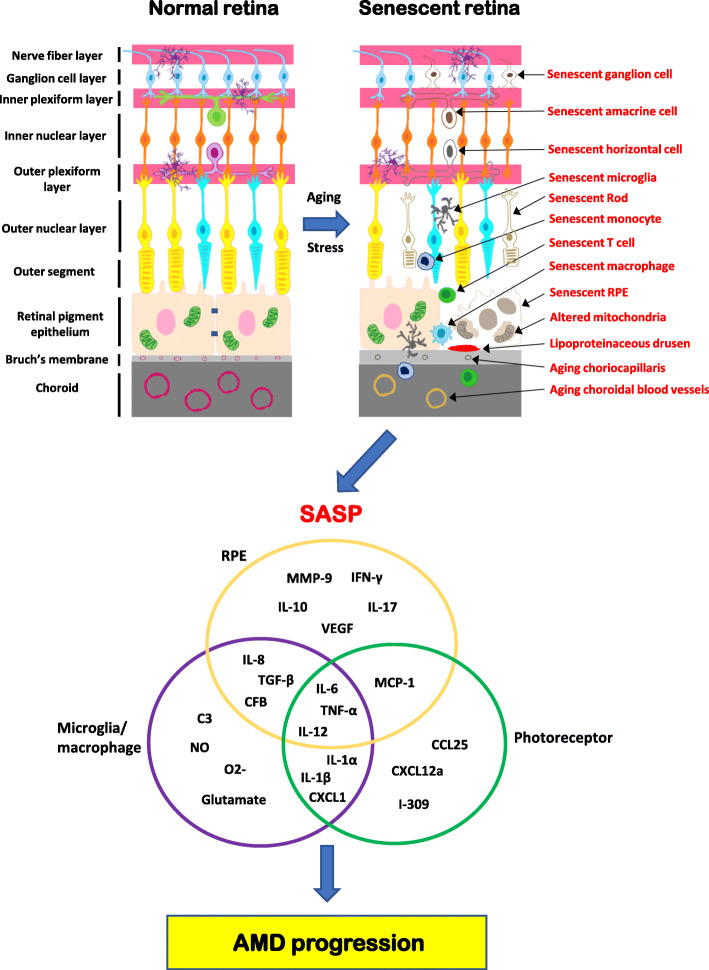
A schematic diagram of consequences of the senescent retina and induction of SASP during progression of AMD. A healthy retina is an immune-privileged ocular tissue, with active immune regulatory networks and immune cell networks supporting normal retinal cell morphology and function [1, 7]. In contrast, a “senescent” retina has both damaged cells and impaired function with alterations in microglial morphology, migration, and infiltration of systemic immune cells. Additionally, resident neuronal cells (ganglion cells, horizontal cells, amacrine cells, and photoreceptors), RPEs, and microglia/macrophages, enter a senescent state in the senescent retina. Drusen begin to accumulate between the Bruch’s membrane and RPE or in the subretinal space (subretinal drusenoid deposits). The Bruch’s membrane thickens and choriocapillaris has a reduced vascular network alongside thinning and diminishing vessels in the aging choroid. Numerous SASP factors, such as IL-6, IL-12, TNF-α, IFN-γ, and IL-8, are released from the senescent retinal cells. ROS, in tandem with damaged DNA, further promote the age-related decline in RPE and photoreceptors resulting in a feedforward cycle of damage. Signaled by the SASP-chemokines released into the tissue environment, immune cells (monocytes, neutrophils, and T cells) extravasate from the blood vessels, infiltrate the retina, and release SASP components, contributing to a chronic inflammation and other AMD-related pathologies

The existence of senescent cells in the human eyes during aging or with AMD is increasingly recognized [23, 65]. The schematic presented in Fig. 2 demonstrates the range of known cell types with associated senescent phenotypes during AMD.

The RPE, besides its role as a barrier cell, is essential for the maintenance of visual cycle and photoreceptor health. In those susceptible to AMD, pathological changes in the RPE frequently occurs prior to the death of rods, followed by cone loss [66]. The functional decline in aging RPE can result in the disruption of the outer blood-retinal barrier (BRB) between the retina and the choroid, which predisposes to drusen deposition and progressive macular damage [47]. Age-dependent accumulation of senescent RPE cells can be detected in human and rhesus monkey eyes, remarkably around the cuticular drusen, displaying alteration in cell morphology and reduced cell density [58, 59]. RPE cells isolated from human aging or AMD donor eyes further highlight the involvement of senescence in AMD, by its expression of senescence-characteristic gene signature, such as upregulation of p16^INK4A^, p21^CIP1^, p53, and bone morphogenetic protein-4 (BMP4) [57, 67, 68]. The epigenetic transcriptome analyses of human AMD-RPE unveiled an association between RPE senescence and the epigenetic dysregulation, such as a differentiation of methylated SKI proto-oncogene, which is a negative regulator of the TGF-β pathway [56]. As a consequence, the over-activated TGF-β signaling induces a SIPS in RPE cells [69] and facilitates an epithelial-mesenchymal transition (EMT), leading to loss of RPE differentiation and compromised cellular function [70]. We have recently demonstrated that RPE cells deficient in interleukin-1 receptor-associated kinase-M (IRAK-M), a key inhibitor of TLR/IL-1R-mediated inflammation, are more susceptible to oxidative stress-induced senescence, evidenced by elevated SA-β-Gal and p21^CIP1^ expression, increased secretion of SASP proteins HMGB1 and IL-6, and decreased nuclear lamina protein LB1. CRISPR-Cas9 editing of IRAK-M gene activation attenuates SIPS and oxidative damage (Liu et al. unpublished data). Reduced expression of IRAK-M has been linked to excessive inflammation, oxidative stress, obesity, and metabolic syndrome, or when autophagy is inhibited in RPE cells [71, 72].

The retina is a neuron-rich tissue that contains more than 60 distinct types of neuronal cells, each playing a specific role in processing visual information [73]. In human neurodegenerative diseases and animal models, neurons of the CNS including cortical, Purkinje, and hippocampal cells, as well as peripheral neurons, display features of cellular senescence and release SASP cytokines [32, 33, 74]. To date, a severe impact of increased senescent neurons has been perceived to exacerbate neurodegeneration in the CNS [32, 65]. In human aging retina, it has been shown that ganglion, amacrine, horizontal and rod cells, but not Müller or astrocyte neuroglia cells, acquire characteristics of cellular senescence [34]. Interestingly, in contrast to the senescent rods identified in aging retina (not AMD), senescent cones are not evident [34]. This finding could explain, at least in part, why the loss of rod photoreceptors exceeds that of cone photoreceptors during the evolution of human AMD [66]. The observations in the aging retina should be nuanced with data obtained from disease settings. For example, premature senescence of retinal ganglion cells in mice can be induced by experimental ocular hypertension (a model of glaucoma), and an early removal of senescent ganglion cells and their SASP secretion in p16-3MR transgenic mice subjected to ocular hypertension prevents disease progression by rescuing surrounding healthy cells [75]. Whilst there is no evidence of astrocyte senescence in retinal diseases, its contribution to age-related neurodegenerative diseases is recognized both in vitro and in vivo [76].

Choroidal blood vessels provide oxygen supply to the highly metabolically active retina, in particular the photoreceptors. One of the earliest detectable events in AMD is the loss of endothelial cells in the choriocapillaris [77]. Data have shown that aging choroidal endothelial cells in rhesus monkeys exhibit high level of SA-β-Gal and aberrant cytoskeletal contractility, which is mediated by upregulated cytoskeletal Rho activity [60]. These age-related changes in choroidal endothelial cells cause choroidal vascular stiffness (loss of flexibility) and sensitize the choriocapillaris to MAC-induced endothelial death and vascular dysfunction [60]. Senescent retinal vascular endothelial cells are also observed in the aging retina and associated with pathogenesis of retinopathy [34]. It is now recognized that senescence of retinal or choroidal vascular endothelium and, as a consequence, vascular dysfunction play an important role in the progression of different retinal diseases, including AMD [60, 78].

The yolk sac-derived retinal microglia are tissue-resident macrophages and the first-line guardian for regional immune surveillance and tissue repair [7]. However, microglia cell senescence may contribute to a persistent inflammation and loss of tissue homeostasis during the development of AMD [61, 79, 80]. In the aged mouse retina, microglia exhibit significant changes in the expression of key genes controlling inflammatory responses including NF-κB, C3, and complement factor B (CFB). This indicates a role of senescent microglia in promoting complement and immune dysregulation [81]. Moreover, using a transgenic reporter line (heterozygous *Cx3cr1*^*+/gfp*^), senescent retinal microglia in the aged retina are morphologically different from healthy microglia, displaying significantly smaller, less branched dendrites, with slower process motility [61]. These morphological changes downplay their functional capacity to survey and interact with the tissue environment, responding to danger-associated triggers and performing phagocytosis. This perturbation can result in the accumulation of neurotoxic debris or diseased cells that further leads to an exaggerated inflammation and metabolic stress [61, 80]. Therefore, this transformation in phenotype induces disordered microglial distribution in the retina, which may precipitate the formation of AMD.

## Senescence in systemic immune cells contributes to retinal degeneration by impaired inflammatory regulation and immune clearance

Age-related alterations in the tissues, including the aging retina, need to be put in context to changes in an aging immune system [82, 83]. Age-related dysregulation and decline in the immune system are termed “immunosenescence,” a process wherein the immune cells display reduced proliferative capacities and altered functions in response to mitogen or antigen stimulation, as frequently observed in aging innate and adaptive immune cells [84, 85]. Whilst molecular determinants for immunosenescence remain undefined, clinical and experimental evidence reveals remarkable similarities and association between immunosenescence and cellular senescence occurred in immune cells, in particular lymphocytes and macrophages exhibiting common senescence markers such as telomere shortening and increased expression of p16^INK4A^, p21^CIP1^, SA-β-Gal, proinflammatory cytokines, and cytotoxic molecules [86–89]. It is noteworthy that while senescence is an overarching phenomenon, it can manifest itself in a cell-specific nature. The known hallmarks of immunosenescence include a reduction in peripheral lymphocyte numbers, a relative increase in frequency of memory CD4+ and CD8+ T cells (especially Th17, but reduced Treg inducibility and stability), a chronic, low-grade inflammation (inflammaging) together with subclinical accumulation of proinflammatory factors, and activation/infiltration of myeloid cells in tissues [5, 82, 90, 91]. The progression of AMD involves active interplays between the innate and adaptive immunity. For example, there is an increase in serum level of C5a in AMD patients, which significantly stimulates the production of IL-22 and IL-17 by T cells [7, 92]. A growing body of evidence has shown that senescence in immune and tissue-resident cells collectively contribute to the development of many disorders in the elderly, such as autoimmune diseases, chronic inflammatory diseases, neurodegeneration, and certain cancers [49, 85, 93].

Innate immune cell types including neutrophils, monocytes/macrophages, and dendritic and natural killer cells have been identified to acquire general senescent characteristics with advanced age, exhibiting dysregulated immune responses and impaired functional capacity [83, 85, 94–96]. Under normal physiological conditions, senescent cells are primarily eliminated by infiltrating leukocytes from the blood or resident immune cells (e.g., microglia), attracted by SASP mediators released from senescent cells. Different immune clearance mechanisms are recognized, such as receptor-mediated phagocytosis or neutrophil extracellular traps (NETs)-mediated NETosis [83, 97]. However, aged phagocytes such as macrophages and neutrophils demonstrate reduced phagocytotic activity and clearance capacity [50, 83]. Conversely, senescent innate immune cells express increased levels of proinflammatory mediators, which further promotes systemic and local inflammation [7, 72, 82]. Further evidence shows that senescent macrophages induce the production of atypical lipid species within the retina, through miRNA-regulated gene transcription [98, 99]. In aged mice, macrophages isolated express high levels of miRNA-33 and 150, which downregulates the expression of ATP-binding cassette subfamily A member 1 (ABCA1) and stearoyl-CoA desaturase-2 (Scd2) (both are regulators in cellular phospholipid and cholesterol homeostasis), therefore resulting in aberrant lipid metabolism and inflammation in aging retina [98, 99]. Of translational relevance, human peripheral blood mononuclear cells isolated from AMD patients express increased miRNA-150 compared with the cells from age-matched healthy donors [99]. These findings imply that targeting the senescent innate immune cells may protect the senescence-driven disturbance in inflammatory and metabolic pathways during degenerative processes of retina.

Senescent T cells increase in frequency in the blood of individuals with infections, autoimmune or age-associated conditions [100, 101]. Senescent T cells are characterized by a unique secretory profile and loss of costimulatory molecules CD28, CD27, and CD40L [100, 102]. The CD56 + CD28− subpopulation of CD8+ T cells increase in the peripheral blood in both dry and wet subsets of AMD and present increased cytotoxic function [103]. Interestingly, increased numbers of cytotoxic CD8+ T cells have been ascertained in the macular choroid of human eyes with drusen [104]. Hitherto, there is yet unequivocal evidence for the role of senescent B cells linked to AMD, nor any significant difference in frequency of any B cell subset between AMD patients and age-matched healthy individuals found [105]. Nevertheless, retina-specific autoantibodies were detected in sera and retinas of patients with early AMD, according to some studies [106, 107].

## Cellular senescence promotes AMD through a network of molecular cascades

Oxidative stress instigated by cigarette smoke concentrate (CSC) or hydrogen peroxide (H_2_O_2_) can induce SIPS in human RPE cells, characterized by increased ROS and DNA lesions, downregulated CFH, and production of VEGF, IL-6, and IL-8 cytokines [53]. Oxidative stress also stimulates the p16^INK4A^/RB pathway responsible for the onset and maintenance of the senescent state. Cell cycling, especially during G1/S transition, is only permitted by the activation of RB pathway through the sequential phosphorylation cascade, which is negatively regulated by p16^INK4A^ that inhibits cyclin A-, cyclin E-, and cyclin D-dependent kinase complexes. Upon adverse or prolonged exposure to oxidative stress conditions, nuclear expression of p16^INK4A^ is elevated. Binding of p16^INK4A^ to cyclins leads to dephosphorylated or hypophosphorylated RB. As a result, the subsequent engagement of the E2F transcription factor is impeded, leading to the cessation of cell division and hence senescence [108].

AMD is strongly associated with the single-nucleotide polymorphisms (SNPs) of high-temperature requirement A 1 (*Htra1*) gene on the chromosome 10q26 AMD locus [1]. The high-risk SNP allele, rs11200638, in the cis-regulatory region of *Htra1*, strengthens transcription of the gene. Indeed, increased HTRA1 protein has been found within drusen of both dry and wet AMD [55]. The HTRA family of serine proteases is pivotal in the maintenance of ATP-independent protein quality and cell function under stress conditions, but excessive expression of HTRA1 can induce RPE senescence, extracellular matrix deposition, and polypoidal choroidal vasculopathy associated with AMD [55, 109]. Oxidative stress has been shown to induce the abnormal transcription and production of HTRA1 in human RPE; the exceptional extracellular level of HTRA1 promotes SIPS in RPE cells via activating p38 MAPK in a HTRA1 protease activity-dependent mechanism [55]. Transgenic mice expressing human HTRA1 in RPE exhibit features of AMD, including RPE atrophy and photoreceptor degeneration. HTRA1-overexpressing transgenic mice aged more than 11 months develop occult CNV, partly through the degradation of elastic lamina of Bruch’s membrane and an increase of VEGF [110].

Endogenous catalysts including telomere erosion, DNA damage, oncogenic activity, or heterochromatin disruption can induce p53-mediated senescence, which is under the regulation of ataxia telangiectasia mutated (ATM) and ataxia telangiectasia and Rad3-related (ATR) kinases for the maintenance of genome integrity. An important downstream molecule of the p53 pathway is p21^CIP1^, which regulates cellular senescence by mediating p53-dependent G1-phase cell cycle arrest [111]. By inhibiting RB phosphorylation, overactivated p21^CIP1^ blocks its binding to E2F. In addition to its role as the guardian of genome integrity, the p53 regulatory machinery is also intensively involved in ROS generation, autophagy, innate immunity [57], and metabolic pathways, which also contribute to senescent changes in the RPE and SASP, as shown by primary human RPE cell cultures [57].

Interestingly, the nutrient-sensing mTOR signaling pathway also plays a part in cellular senescence [78, 112]. Dysregulated mTORC1 activity is linked with aging, and inhibition of mTOR signaling has been shown to confer extended lifespan and autophagy repression in myriad of animal models [113]. The two different signaling complexes mTORC1 (mTOR/Raptor/GβL) and mTORC2 (mTOR/Rictor/GβL/SIN1) are both functional in the human eyes. Adenosine monophosphate-activated protein kinase (AMPK) behaves as a metabolic checkpoint that inhibits cellular growth by suppressing the mTORC1 signaling pathway [114]. Treatment with low dose of rapamycin, the specific mTORC1 inhibitor, can inhibit RPE senescence in vitro, evidenced by a decreased percentage of senescence cells and decreased SA-β-Gal activity. The mTOR pathway in human RPE cells with high population doubling (PD) number responds more sensitively to exogenous nutrient stimuli such as amino acids than the cells of lower PD number [112]. The retina is recognized as the most metabolically active tissue in the human body, and its high mitochondrial activity and energy demands confer vulnerability to nutritional insults and metabolic dysregulations. In vivo, mTOR inhibition by rapamycin can prevent photoreceptor degeneration induced by RPE stressor exposure [115] or through RPE-specific mTORC1 activation [116]. RNA-Seq analyses on RPE of human AMD eyes revealed an overactivation of the mTOR pathway, hyperphosphorylation of AMPK, and the significant changes in its target pathways. As a result, deranged profiles of metabolites derived from glycophospholipid, lipid, and protein metabolism were detected by metabolomics and lipidomics [24]. This indicates that the mTOR network not only regulates metabolism and aging in response to nutrition and growth factors but is also associated with the acquisition of a senescent phenotype [112]. A caveat is that data to mechanistically link mTOR overactivation with senescence in AMD are lacking.

As a consequence of cellular senescence, SASP also plays a proactive role in the reinforcement of senescence in an autocrine or paracrine manner [23, 27, 117]. SASP can induce a low-grade inflammation, drive cellular senescence of adjacent cells, and display detrimental effects in the tissue microenvironment, associated with the development of age-related pathologies [117]. For example, in an oxygen-induced retinopathy (OIR) model, cellular senescence initially observed in the retinal ganglion neurons propagates to other cell populations of the retina (microglia and vascular endothelial cells), further contributing to the retinopathy [27]. Initiation and maintenance of SASP require the transcription factor, GATA-binding protein 4 (GATA4), which is activated by ATM and ATR (two key protein kinases acting as DNA damage sensors) and actuates NF-κB signaling pathway [118].

SASP arising from chronic senescence is a master and protracted source of the chronic inflammation typical in aging. It generates SASP-congruent inflammatory mediators detrimental to surrounding cells and tissue [119]. SASP is therefore considered a potential target for treating age-associated diseases, notwithstanding the fact that the composition of SASP differs in accordance with diseases, triggering factors and cell types [65]. To date, mainly in vitro studies of human RPE cells under RS or SIPS conditions (e.g., age-related DAMPs, Aβ, CSC, or oxidized photoreceptor outer segments) have revealed a panel of RPE-related SASP genes or proteins, including IL-6, IL-8, IL-10, IL-12, IL-17, MCP-1, TNF-α, VEGF, MMP-9, IFN-γ, CFB, and TGF-β [53, 120–122] (Fig. 2). Senescent retinal microglia, systemic or infiltrating monocytes/macrophages associated with AMD, have been found to express escalated inflammatory factors IL-1α, IL-1β, IL-6, IL-8, IL-12, TNF-α, C3, CFB, CXCL1, TGF-β, nitric oxide (NO), superoxide anion (O2-), and glutamate [65, 81, 123, 124]. Circumstantial evidence from murine models of diabetes-induced retinopathy and defects in lipoprotein receptor indicates inflammatory secretion of IL-1α, IL-1β, IL-6, IL-12, TNF-α, MCP-1, CCL25, CXCL1, CXCL12a, and I-309 by abnormal photoreceptors [125, 126]. Of note, such proinflammatory mediators derived from altered photoreceptors are all recognized or reported SASP components in other cell types or tissues [127–130]. The involvement of SASP in AMD has clinical manifestation too, as a number of inflammatory cytokines have been uncovered to be elevated either systemically in the sera or locally in the aqueous humor of patients with AMD, particularly IL-6, IL-8, IL-12, MCP-1, TNF-α, IL-1α, IL-1β, and IL-17 [82, 123, 131]. Additionally, IL-22 and IL-17, primarily produced by Th17 subset of T cells, were found to have higher levels in the sera of patients with wet AMD, while increased IL-17 level also detected within AMD lesions [132].

Recent data demonstrate that cellular senescence, autophagy dysfunction, and abnormal inflammatory response are interlinked processes that exert a cause-and-effect on each other, a vicious cycle ultimately leading to retinal degeneration. In RPE cells, the interaction between nuclear factor erythroid 2-related factor 2 (Nrf2) and p62 proteins enhances autophagy and improves Nrf2-mediated antioxidant response to dampen inflammation and protect against oxidative stress. Cellular senescence triggers the downregulation of Nrf2 and weakens the interaction between Nrf2 and p62 [78, 133]. Consequently, the normal function of autophagy is hindered, predisposing the RPE to abnormal inflammation. Dysfunctional autophagy impairs the clearance system, precipitating the formation and accumulation of lipofuscin and drusen. Lipofuscin and drusen deposition accelerate cellular senescence and drive active inflammatory responses, which can further promote autophagy dysfunction [78, 117].

Cellular senescence also coordinates structural and functional modifications in multiple cellular organelles. Among them, mitochondria and lysosomes play central roles in metabolic reprogramming and regulation of autophagic clearance the key mechanisms underlining senescence responses [134, 135]. Not only is mitochondrial alteration a characteristic feature of senescence, but defects in various mitochondrial pathways are noxious insults eliciting entry into senescence. In addition to excessive production of ROS by complex I and III, mitochondrial stress signals can equally arise from mitochondrial dynamics (fission and fusion), electron transport chain, redox state, mitochondrial metabolites, and calcium homeostasis, especially in tissues such as the retina where a healthy mitochondrial turnover is requisite for normal function [136, 137]. In a healthy eye, the retina functions as a “metabolic ecosystem,” where the RPE primarily exploits mitochondrial oxidative phosphorylation (OXPHOS) for energy production and transports glucose to the photoreceptors that mainly rely on aerobic glycolysis (Warburg effect) [138]. In vivo RPE metabolic shift from OXPHOS to glycolysis causes RPE dysfunction and subsequent photoreceptor death [139]. Aged human RPE exhibits depleted reserve capacity of mitochondrial respiration that generates additional ATP in combating stress, leading to increased oxidative stress-induced cell death [140]. Metabolomics analyses of AMD-derived RPE reveal aberrant metabolites of mitochondrial bioenergetics [24]. In senescent cells, including senescent RPE, altered mitochondrial metabolism and membrane potential promote inflammation via assorted pathways such as ROS and ATP production, DAMP (e.g., mtDNA) stimulation, and calcium exchange [141–144].

Mitochondria alongside lysosomes execute metabolic tuning to guide the whole-cell responder program in response to cellular stressors. Recently, a mitochondrial-lysosomal axis has been proposed as a hinge in the control of cellular senescence [135, 145]. Diminished lysosomal activities and perturbation in its communication with mitochondria are associated with age-related pathologies [134, 135, 145]. Age-associated senescent cells frequently exhibit disturbance in lysosome-mediated autophagy flux, which results in altered cellular proteostasis and accumulation of lipofuscin, an autofluorescent lipid-rich protein aggregate with multiple metabolic origins [146, 147]. In both atrophic and neovascular AMD, accumulation of RPE intracellular lipofuscins and lysosomal destabilization-induced NLRP3-inflammasome activation occur, inferring lysosomal damage [147, 148]. Lysosomal dysfunction is shown to impact on the mitophagy-regulated mitochondrial turnover, leading to increased mitochondrial mass and superoxide formation, and in turn, altered mitochondrial metabolism and fission aggravate lysosomal impairment and lipofuscinogenesis [145, 149]. Pertinently, in vitro data suggest that mitochondrial dysfunction plays an initiating role, while lysosomal dysfunction is more directly responsible for autophagy impairment and cellular senescence [150]. Murine models with deficient expression of proteins involved in mitochondrial biogenesis (PGC-1α), antioxidant response (NFE2L2), or lysosomal acidification (CRYBA1) demonstrate alleviated RPE mitophagy, dysmorphic photoreceptor changes, and reduced vision [151, 152]. Not surprisingly, therefore, targeting the mitochondrial-lysosomal axis might constitute new strategies for anti-aging intervention.

## The repertoire of senotherapeutic strategies is expanding to fight a spectrum of diseases involving senescence

Teasing apart the distinct mechanisms of cellular senescence from physiological aging has driven the development of therapeutic approaches that target chronic senescence for the treatment of age-related diseases. The core principles of these strategies are (1) selective clearance of senescent cells by genetic/epigenetic modulation or small compounds to counteract the apoptosis-resilience of senescent cells and (2) blockade of the proinflammatory SASP component [119].

Senolytics represent a class of small molecules that are being developed to treat a number of aging-associated pathologies such as diabetes, frailty, cardiovascular disease, and cancers [153]. Research is focused on approaches that exploit the relative resistance to apoptosis against both intrinsic and extrinsic pro-apoptotic signals displayed by senescent cells [119], conferring the pro-survival advantage to persist under stress conditions [154]. Therefore, targeting these pathways may permit selective elimination of senescent cells and limit any detrimental side-effects on healthy cells [154]. The first published senolytic was a cocktail of dasatinib, a pan-tyrosine kinase inhibitor, and quercetin, a naturally occurring flavonoid [155]. When orally administered in combination, dasatinib and quercetin (D + Q) in murine models were shown to selectively reduce senescent cells in osteoporosis [153]. Two recent clinical trials evaluating D + Q further demonstrate protective effects in patients with idiopathic pulmonary fibrosis [156, 157]. Treatment led to a reduction in senescent cell burden [156], evidenced by reduced p16^INK4A^ and p21^CIP1^-expressing adipocytes, reduced SA-β-Gal activity in skin biopsy tissues, and decreased levels of circulating SASP factors including IL-1α, IL-6, and MMP-9 and MMP-12. However, treatment also resulted in unwanted side-effects, including delayed wound healing, neutrophilia, and thrombocytopenia, highlighting how long-term use of senolytic agents requires careful consideration, particularly when chronic dosing is typically required for treatment of age-related disorders [158].

The p53/p21^CIP1^ axis could also be a promising target for senolytic agents [159]. p53 is the most frequently mutated tumor suppressor gene in human cancer, and its transcriptional activity controls a myriad of biological processes—transient cell cycle arrest, senescence, and apoptosis [160]. In vivo studies show that a modified FOXO4/p53-interfering peptide abates liver chemotoxicity induced by doxorubicin and restores renal function in progeroid and naturally aged mice [159]. p21^CIP1^, a primary transcriptional target of p53, is necessary for the survival of senescent cells [161], as demonstrated by the alleviation of liver cell senescence and liver fibrosis in p21^CIP1^-deficient mouse models [162]. Further human research in the development of FOXO4 peptide is needed to target the p53/p21^CIP1^ pro-survival pathway of senescent cells.

Targeted immune cell attack to senescent cells could also show both specificity and efficiency. Senescent cells upregulate the immune recognition cell surface receptor natural killer group 2D (NKG2D), a marker not usually expressed by normal cells. Selective expression of NKG2D on target cells may facilitate immunotherapeutic approaches designed to eliminate these cells [163], and approaches developed for cancer treatment are being investigated for senotherapeutics. For example, inhibiting the immune checkpoint inhibitors such as programmed death 1 (PD-1) is a powerful treatment of cancer and has also been shown to ameliorate symptoms of AD in murine models [164]. In addition to immune-boosting strategies, chimeric antigen receptor (CAR) T cells may provide an option to redirect immune responses against senescent cells [165].

Although the elimination of senescent cells appears to be a favorable strategy, it may compromise some beneficial aspects of cellular senescence or even prevent the antitumor protection it affords. Interventions designed to disrupt the proinflammatory microenvironment and suppress SASP secretion provide an alternative route leading to senotherapy [153, 166]. Contrary to senolytics that selectively eliminate senescent cells, senomorphic agents target proinflammatory signaling networks [49]. Several signaling pathways that converge and activate the NF-κB and C/EBPβ pathways are amenable to modulation by a broad spectrum of drugs that are already approved by the FDA [167]. For instance, IL-1α/IL-1 receptor signal transduction is upstream of NF-κB, and the use of neutralizing antibodies against either IL-1α or its receptor is sufficient to reduce NF-κB transcriptional activity [167]. mTOR inhibitors, such as rapamycin and its analogs, can abolish SASP by reducing the expression of membrane-bound IL-1α [168]. Clinical trials of the AMPK activator metformin, another potential senomorphic, are currently ongoing or completed to treat osteoporosis and disc degeneration in elderly people, with encouraging success [49].

While limiting the extent of inflammation is potentially promising, using strong anti-inflammatory drugs can itself elicit a range of side effects; hence, targeting specific components of SASP could provide a safer alternative to mitigate the deleterious effects of SASP. Cytokines, such as IL-6, IL-8, and components of SASP, could serve as possible targets. A plethora of monoclonal antibodies are already commercially available, which could potentially be used to neutralize SASP. Approved drugs such as siltuximab or tocilizumab could address the issue of senescence by blocking IL-6 or its receptor. These drugs have already been launched for the treatment of life-threatening cytokine release syndrome (CRS) as well as IL-6-driven rheumatic and malignant diseases [169, 170].

## Antagonizing senescence in the eye opens a therapeutic window for AMD

**Fig. 3:**
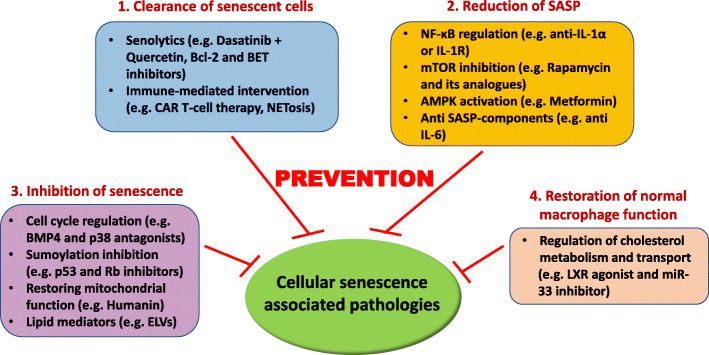
Prospective senotherapeutic strategies to subvert AMD progression. (1) Selective elimination or modulation of senescent cells through senolytics such as dasatinib + quercetin, inhibitors of Bcl-2 or BET proteins, chimeric antigen receptor (CAR) T cells, or by modulating neutrophil-induced NETosis, which can redirect immune responses against senescent cells. (2) Modulation of senescence-related signaling networks (senomorphics) to attenuate SASP through, for example, regulating NF-κB, mTOR, and AMPK pathways, or utilizing monoclonal antibodies against SASP factors, such as IL-6. (3) Inhibition of senescence in the eye using cell cycle regulators, p53/Rb sumoylation inhibitors, mitochondria-derived peptides, or lipid mediators. (4) Restoration of cholesterol homeostasis/flux and normal function of macrophages via liver X receptor (LXR) agonists or miR-33 inhibition, which has proved beneficial in the inhibition of retinal degeneration in vivo

To address AMD from the perspective of senescence, therapeutic concepts and attempts based on pre-clinical animal models and clinical trials are mainly designed to prevent senescence cell burden, eliminate senescent cells, inhibit harmful SASP secretion, and restore autophagic housekeeping (Fig. 3).

There is evidence indicating that both senescence and SASP directly contribute to cell loss and subsequent accumulation of ROS-producing deposits, together accelerate retinal dysfunction. Therefore, development of senolytic small molecules that selectively target senescent cells are directed to diseases of the aging eye [65]. For example, certain B cell lymphoma 2 (Bcl-2) family proteins are upregulated in senescent cells to evade apoptosis. Selective targeting of Bcl-2 proteins using inhibitory drugs UBX1325 and UBX1967, to promote apoptosis in senescent cells, is showing promise for the treatment of age-related ocular diseases including AMD. It will be interesting to see what results come from the recently initiated phase I study to evaluate safety, tolerability, and the pharmacokinetics of intravitreal delivered administration of UBX1325 in patients diagnosed with diabetic macular edema [65, 171]. Additionally, synthesized small compounds targeting other pro-survival pathways, such as inhibitors of bromo and extraterminal domain (BET) proteins, have been identified via high-throughput screening and show promising therapeutic value as they attenuated RPE cell loss in vitro and protected ganglion cells in vivo [172, 173].

Retinal and choroidal blood vessels, compared with other parts of the eye, have greater remodeling capacity and have served as an effective therapeutic target in neoangiogenic retinal diseases [97, 174]. Elimination of senescent blood vessels may also lead to beneficial vascular remodeling and tissue homeostasis. In the OIR mouse model, age-independent senescence activation in retinal vasculature elicits a senescent secretome and innate immune response characterized by neutrophil recruitment from the blood [97]. The infiltrating neutrophils, releasing neutrophil extracellular traps (NETs), eliminate pathological senescent vasculature through an alternative mechanism of cell death termed as NETosis, thereby promoting retinal vascular regeneration and tissue repair [97]. The findings highlight that the immune cell-mediated clearance of senescent vascular cells ameliorates the severity of pathological neovascularization. More broadly, these immunotherapies via manipulating the sterile inflammation could potentialize a senotherapeutic strategy in other retinal diseases such as AMD [1, 7, 83].

Oxidative stress-induced cellular senescence is another important contributing factor in the development of AMD [39]. Oxidative stress-induced pathological changes in senescent cells highlight other potential targets for senotherapeutics. As a member of TGF-β superfamily, BMP4 is the key regulator of RPE morphogenesis and proliferation [67, 175]. Elevated BMP4 expression levels are observed in RPE cells in response to oxidant exposure and at the macular RPE from patients with dry AMD. In primary human RPE cells, overexpression of endogenous BMP4 or the addition of exogenous BMP4 triggers increased expression of cell-cycle checkpoint proteins and cellular senescence, via activation of Smad, p38, and p53/p21^CIP1^, and decreased RB phosphorylation [67]. The premature senescence of RPE, induced by either oxidant exposure or overexpression of BMP4, can be reversed using BMP4 antagonists, such as Chordin-like 1 protein or p38 inhibitor SB203580. Additionally, higher levels of circulating BMP4 is detected in serum samples of wet AMD donors compared to age-matched controls. In collaboration, BMP4 treatment of RPE cells mediates the activity of MMPs, disrupts the barrier integrity of RPE, and induces their migration [176]. The preferential expression of BMP4 in AMD may represent a potential target to selectively protect against oxidative stress and senescence-associated AMD pathology.

Recent work suggests that sumoylation may represent another novel therapeutic target for the treatment of AMD [177]. Sumoylation is a critical process that regulates retina and RPE aging and refers to a reversible post-translational modification that involves the conjugation of small ubiquitin-like modifier (SUMO) to a target protein. As discussed, p53 and RB are implicated in RPE senescence, and sumoylation modulates the function of p53 and RB by regulation in their transcriptional activity, target-binding, stability, and protein turnover. Sumoylation of chaperon proteins such as Hsp70 can also facilitate RPE senescence through blunting autophagic clearance [178]. The inhibition of sumoylation has been demonstrated to ameliorate proinflammatory cytokine secretion in oxidative stress-induced RPE senescence of aging mice [177].

Restoring mitochondrial homeostasis serves to protect cells against senescence. Within a healthy retinal “metabolic ecosystem,” RPE cells primarily exploit mitochondria-dependent OXPHOS for energy production and transport glucose to photoreceptors that mainly rely on aerobic glycolysis [138]. However, aged RPE and RPE derived from AMD patients demonstrate impaired mitochondrial activity that directs a metabolic shift aerobic glycolysis, depleting photoreceptors of energy supply [24]. There are strategies to support mitochondrial function in RPE and counteract the aging process and progress of AMD [179]. Humanin, one of the mtDNA-encoded peptides, protects mitochondria and maintains cell homeostasis and health in experimental models of age-related diseases including tumor and neurodegeneration [180]. In human primary RPE cells, humanin improves mitochondrial biogenesis and activities, restricting oxidative stress-induced senescence [54].

The aggregation of Aβ is recognized as the prominent driver of AD, and in the eye, Aβ is an important component of drusen in AMD, inducing inflammatory responses and loss of photoreceptors. Elovanoids (ELVs) are a newly identified class of lipid mediators synthesized in RPE and have been shown to protect photoreceptors against oxidative stress [181]. Furthermore, ELVs (particularly ELVs with 32 and 34 carbons) have been demonstrated to counteract oligomeric Aβ (OAβ, a highly cytotoxic form of Aβ species)-induced senescence and inflammatory gene transcription in primary human RPE cells [182].

Aging and senescence are also sensed by retina-resident or infiltrating macrophages. Altered polarization of macrophages and their secretory profiles are involved in the pathologic angiogenesis and formation of a proinflammatory milieu during AMD development [1, 7, 8, 183]. The regulation of macrophage function is under the influence of lipid metabolism and transport [184]. Impaired cholesterol efflux has been associated with reduced drusen scavenge capacity and atypical activation in senescent macrophages [184]. The liver X receptor (LXR), a member of nuclear receptor family of transcription factors, is one of the activators of ABCA1 that regulates cholesterol efflux of macrophages. Interestingly, monocytes from older humans and macrophages from AMD donor eyes showed reduced ABCA1 expression [98]. Macrophage-specific ABCA1-deficient mice exhibit increased intracellular deposition of free cholesterol and abnormal alternative activation of macrophages, promoting accelerated retinal aging and degeneration. Restoration of cholesterol homeostasis using LXR agonist or inhibitor of miR-33, which regulates the expression of genes involved in cellular cholesterol metabolism including ABCA1, reverses the accelerated retina aging in the mice [98].

## Conclusions

The nascent field of senolytics targeting age-related illness including AMD is exciting. Compared to the existing approaches, senotherapeutics target a potential root cause of AMD progression. However, we need to further understand how cells enter senescence and how the beneficial adaptive senescent response shifts to a deleterious chronic senescence. In order to develop therapies to prevent AMD progression, it remains crucial to identify a retinal-specific pattern of senescence and SASP, identify which cells involved in AMD are susceptible and when they enter senescence, and identify an AMD-related SASP secretion profile. To be successful and sustainable, any development of therapy needs to be specific and reduce off-target or potential oncogenic effects and couple senotherapy with an efficient drug delivery essential to ensure bioavailability and selective targeting, as well as controlled drug release with minimized dosing frequency.

## Data Availability

All data generated or analyzed during this study are included in this published article.

## Abbreviations

ABCA1: ATP-binding cassette subfamily A member 1
Aβ: Amyloid beta
AD: Alzheimer’s disease
AMD: Age-related macular degeneration
AMPK: Adenosine monophosphate-activated protein kinase
AREDS: Age-Related Eye Disease Study
ATM: Ataxia telangiectasia mutated
ATP: Adenosine triphosphate
ATR: Ataxia telangiectasia and Rad3-related
Bcl-2: B cell lymphoma 2
BET: Bromo and extraterminal domain
BMP4: Bone morphogenetic protein 4
BRB: Blood-retinal barrier
CAR: Chimeric antigen receptor
CCL25: C-C motif chemokine ligand 25
CDK: Cell cycle activators
C/EBPβ: CCAAT-enhancer-binding protein beta
CFB: Complement factor B
CFH: Complement factor H
CFI: Complement factor I
CNS: Central nervous system
CNV: Choroidal neovascularization
CRS: Cytokine release syndrome
CRYBA1: Crystallin beta A1
CSC: Cigarette smoke concentrate
CXCL1: CXC chemokine ligand 1
CXCL12a: CXC chemokine ligand 12a
DAMPs: Damage-associated molecular patterns
DPS: Developmentally programmed senescence
DR: Diabetic retinopathy
DSBs: Double-strand breaks
D + Q: Dasatinib and quercetin
ELVs: Elovanoids
EMT: Epithelial-mesenchymal transition
FOXO4: Forkhead box O4
GβL: G protein beta subunit-like
GATA4: GATA binding protein 4
HMGB1: High mobility group box 1
HTRA: High-temperature requirement A
IFN-γ: Interferon gamma
IL-1R: Interleukin-1 receptor
IRAK-M: Interleukin-1 receptor-associated kinase-M
LCA: Leber congenital amaurosis
LXR: Liver X receptor
MAPK: Mitogen-activated protein kinase
MAC: Membrane attack complex
MCP-1: Monocyte chemoattractant protein-1
MMP-9: Matrix metallopeptidase-9
mTOR: Mammalian target of rapamycin
mTORC1 or 2: Mammalian target of rapamycin complex 1 or 2
NETs: Neutrophil extracellular traps
NF-κB: Nuclear factor kappa-light-chain-enhancer of activated B cells
NFE2L2: Nuclear factor, erythroid 2 like 2
NKG2D: Natural killer group 2D
NLRP3: NOD-, LRR-, and pyrin domain-containing protein 3
NO: Nitric oxide
Nrf2: Nuclear factor erythroid 2-related factor 2
OAβ: Oligomeric amyloid beta
OIR: Oxygen-induced retinopathy
OSKM: Transcription factors OCT4, SOX2, KLF4, and MYC
OXPHOS: Oxidative phosphorylation
O2-: Superoxide anion
PD: Parkinson’s disease
PD: Population doubling
PD-1: Programmed death-1
PGC-1α: Peroxisome proliferator-activated receptor gamma coactivator 1-alpha
RB: Retinoblastoma protein
ROS: Reactive oxygen species
RPE: Retinal pigment epithelium
RS: Replicative senescence
SA-*β*-Gal: Senescence-associated *beta* galactosidase
SAHF: Senescence-associated heterochromatic foci
SASP: Senescence-associated secretory phenotype
Scd2: Stearoyl-CoA desaturase-2
sFlt-1: Soluble fms-like tyrosine kinase-1
SIN1: Stress-activated protein kinase-interacting protein
SIPS: Stress-induced premature senescence
SNPs: Single-nucleotide polymorphisms
SUMO: Small ubiquitin-like modifier
TGF-β: Transforming growth factor beta
TLR: Toll-like receptor
TNF-α: Tumor necrosis factor alpha
VEGF: Vascular endothelial growth factor
XLRS: X-linked retinoschisis
